# Genetic variant I148M in *PNPLA3* is associated with the ultrasonography-determined steatosis degree in a Chinese population

**DOI:** 10.1186/1471-2350-13-113

**Published:** 2012-11-23

**Authors:** Yiling Li, Chao Xing, Zhong Tian, Hung-Chih Ku

**Affiliations:** 1 Department of Gastroenterology, First Affiliated Hospital of China Medical University, Shenyang, 110001, China; 2 McDermott Center for Human Growth and Development, University of Texas Southwestern Medical Center at Dallas, Dallas, TX, 75390, USA; 3 Department of General Surgery, Shengjing Hospital of China Medical University, Shenyang, 110001, China

**Keywords:** *PNPLA3*, NAFLD, Ultrasonography, Steatosis grade

## Abstract

**Background:**

Nonalcoholic fatty liver disease (NAFLD) is an escalating medical problem worldwide. A nonsynonymous single nucleotide polymorphism rs738409 (I148M) in patatin-like phospholipase domain-containing protein 3 (*PNPLA3*) predisposes susceptibility to NAFLD; however, its association with steatosis grade is inconsistent in the literature. In particular, there was no significant association found between I148M and steatosis grade in two East Asian-based studies. In this study we aim to investigate whether I148M is associated with the ultrasonography-determined steatosis degree in Chinese adults.

**Methods:**

203 NAFLD cases and 202 matched controls were recruited. Cases were classified into mild, moderate and severe fatty liver by ultrasonography. Association between I148M and the ultrasonography-determined steatosis degree as well as other clinical parameters was evaluated.

**Results:**

The I148M variant was associated with the ultrasonography-determined steatosis degree with the M allele frequencies being 0.32, 0.54, and 0.87 in mild (n=105), moderate (n=83), and severe (n=15) cases, respectively (*P*–value = 7.6×10^-8^). We also confirmed the interaction between I148M variation and body mass index towards elevated plasma alanine aminotransferase levels in cases (*P*–value = 4.4×10^-4^).

**Conclusion:**

The *PNPLA3* I148M variant is associated with the ultrasonography-determined steatosis degree in Chinese population.

## Background

Nonalcoholic fatty liver disease (NAFLD) is an escalating medical problem worldwide. It affects 20-34% of the population in Western countries [1]; although the prevalence of NAFLD is somewhat lower in East Asia—25% in Japan [2], 18% in South Korea [3], and 15% in China [4]—its incidence has increased rapidly with a growing prevalence in young generations during the last two decades [5,6].

NAFLD is a multifactorial disorder associated with obesity, insulin resistance, type 2 diabetes, and dyslipidemia [1,7]. Familial clustering of steatosis, nonalcoholic steatohepatitis, and cryptogenic cirrhosis suggests that genetic factors contribute to the susceptibility to NAFLD [8,9]. Multiple genes have been reported to be associated with NAFLD by candidate gene studies (for a review see [10]), and, more recently, by genome-wide association studies [11-13]. A nonsynonymous sequence variation (rs738409) in patatin-like phospholipase domain-containing protein 3 (*PNPLA3*) that substitutes methionine for isoleucine at residue 148 (I148M) was first reported to be associated with hepatic triglyceride content in a genome-wide screen [11]; subsequently its association with NAFLD was confirmed in multiple ethnic and geographic groups (for a review see [14]) including the East Asian populations [15-19]. The I148M variant was also reported to be associated with the histological severity of NAFLD (for a review see [20]); however, in two East Asian-based studies, there was no significant association between I148M and steatosis stage [15,16]. In particular, in a sample of 112 Chinese NAFLD patients I148M was found to be associated with fibrosis but not steatosis grade [16]. In this study we investigated the association between I148M and the ultrasonography-determined steatosis degree in a larger collection of Chinese NAFLD patients.

Although the *PNPLA3* I148M variant is not associated with body-mass-index (BMI) in the general population [11,13], it was shown that morbid obesity exposed the association between the 148M allele and plasma alanine aminotransferase (ALT) levels in both obese adults [21] and children [22,23]. Further it was shown in obese children the association between I148M and ALT levels was due to the interaction with abdominal fat [24]. More recently it was shown the 148M allele interacted with obesity towards type 2 diabetes susceptibility [25]. In this study we also attempted to replicate the interaction between I148M and BMI on ALT levels and to investigate whether it contributes to NAFLD susceptibility.

## Methods

A total of 203 unrelated adults with NAFLD were recruited from an outpatient liver clinic at the First Affiliated Hospital of China Medical University in Shenyang, China, between October 2010 and April 2011. The patients were confirmed to have hepatic steatosis by liver ultrasonography and classified into three categories—mild, moderate, and severe—according to established criteria [26]. In particular, five criteria were used to diagnose NAFLD: 1) diffuse enhancement of near field echo in the hepatic region and gradual attenuation of the far field echo; 2) unclear display of intra-hepatic lacuna structure; 3) mild to moderate hepatomegaly with a round blunt border; 4) reduction of blood flow signal in the liver; and 5) unclear or non-intact display of envelop of right liver lobe and diaphragm. Patients meeting criterion 1 and any one of criteria 2-4 were classified as mild; patients meeting criterion 1 and any two of criteria 2-4 were classified as moderate; and patients meeting criteria 1, 5, and any two of criteria 2-4 were classified as severe [26]. All the examinations were performed by one experienced radiologist, who was unaware of the patients’ clinical details and laboratory findings, using a GE Vivid7 ultrasound machine (GE Healthcare, Horten, Norway) equipped with a GE 4C curved array transducer (GE H4904PC). Secondary causes of steatosis—ethanol intake, total parenteral nutrition, hepatitis B and Hepatitis C virus, autoimmune liver disease, hemochromatosis, alpha1-antitrypsin deficiency, Wilson’s disease, use of drugs that promote steatosis—were ruled out. A total of 202 ethnicity-matched controls with normal liver enzyme levels and no steatosis by ultrasonography were recruited from primary care outpatient clinics at the same institution. Written informed consent was obtained using a protocol approved by the ethics committee of the First Affiliated Hospital of China Medical University.

Age and sex were self-reported. BMI was calculated according to the measured height and weight at the time of recruitment. Venous blood samples were obtained from the subjects after an overnight fast (12 hours). Plasma ALT, aspartate aminotransferase (AST), γ-glutamyltransferase (GGT), fasting blood glucose (FBG), triglycerides, high density lipoprotein cholesterol (HDL-C) and low density lipoprotein cholesterol (LDL-C) levels were measured using an automated analyzer. Genomic DNA was extracted from leukocytes using AxyPrep Whole Blood Genomic DNA Miniprep Kit (Axygen Biosciences, USA). The I148M variant was genotyped using a TaqMan assay on a 7900HT Fast Real-Time PCR instrument (Applied Biosystems, Foster City, CA) at 50°C for 2 min, 95°C for 10 min, and then 40 cycles of 95°C for 15 sec and 60°C for 1.5 min. Hardy-Weinberg equilibrium (HWE) was examined by Pearson’s *χ*^2^ goodness-of-fit test in cases and controls, respectively.

Comparisons of demographic and clinical features between cases and controls were performed by a two-sample *t*-test for continuous variables and by a proportion test for categorical variables. Comparisons between caterogies of cases were performed by analysis of variance. Linear regression models were fit to test association between genotype and continuous phenotypes, and logistic regression models were fit to test association between genotype and categorical phenotype. Bonferroni correction was performed to correct for multiple testing. The genotypic value was coded in an additive manner, i.e., 0, 1, and 2 denoted II, IM, and MM genotypes, respectively. The interaction between I148M and BMI for a continuous trait—ALT—was tested by examining the interaction term in a linear regression model; the interaction for an ordinal trait—the ultrasonography-determined steatosis degree—was tested by examining the interaction term in an ordered logistic regression model; the interaction for a binary trait—NAFLD—was tested either by examining the interaction term in a logistic regression model using both cases and controls, or by examining the association between I148M and BMI using only cases. We estimated the power to detect interaction *a priori* given the sample size; when there was no significant interaction detected, we also estimated sample sizes required to achieve the power of 0.8. The power analyses were performed for a quantitative trait [27] and for a binary trait in both case-control [28,29] and case-only [30] designs. All analyses were performed using R [31] except that power analysis in a case-control design was performed using POWER V3.0 [28,29].

## Results

The comparisons of demographic, clinical, and genetic characteristics between the 203 NAFLD cases and 202 controls were reported elsewhere [17]. Here we focused on comparing the characteristics among cases with the different ultrasonography-determined steatosis degrees, as summarized in Table 1. Based on liver ultrasonography the 203 NAFLD cases were classified into three categories—mild degree (n = 105), moderate degree (n = 83), and severe degree (n = 15). The 15 severe cases were on average younger than the mild and moderate cases (38.9 ± 13.9 years versus 47.6 ± 13.3 and 47.4 ± 13.2 years, respectively). BMI increased with the ultrasonography-determined steatosis degree (*P* < 0.01). Liver enzyme levels ALT and AST increased with the steatosis degree (*P*-values < 0.01) whereas GGT levels did not (*P* > 0.10). Triglycerides levels showed a marginal increase with the increase of the steatosis degree (nominal *P*-value = 0.03), whereas LDL-C and HDL-C levels did not (*P*-values > 0.10). FBG levels were higher in the 15 severe cases, though not statistically significant (*P*-value > 0.10).

**Table 1:** **Demographic, clinical, and genetic characteristics of NAFLD cases stratified by the ultrasonography-determined steatosis degree**^†^

**Variable**	**Steatosis grade**			***P*-value**^**‡**^
	**Mild**	**Moderate**	**Severe**	
Sample size	105	83	15	
Age (years)	47.6 ± 13.3	47.4 ± 13.2	38.9 ± 13.9	0.06
BMI (kg/m^2^)	26.1 ± 3.7	27.2 ± 4.4	30.5 ± 5.3	< 0.01
ALT (IU/L)	30.9 ± 21.3	50.1 ± 36.1	106.3 ± 78.5	< 0.01
AST (IU/L)	28.8 ± 26.9	29.5 ± 13.4	59.8 ± 62.46	< 0.01
GGT (IU/L)	63.0 ± 83.8	55.8 ± 48.6	78.2 ± 47.7	> 0.10
Triglycerides (mmol/L)	1.8 ± 1.3	2.3 ± 1.5	2.4 ± 1.8	0.03
LDL-C (mmol/L)	3.4 ± 1.0	3.3 ± 1.1	3.1 ± 1.2	> 0.10
HDL-C (mmol/L)	1.3 ± 0.4	1.4 ± 0.8	1.1 ± 0.2	> 0.10
FBG (mmol/L )	6.0 ± 1.3	6.0 ± 1.1	6.7 ± 1.9	> 0.10
PNPLA3-I148M				
II	52	18	0	7.6×10^-8^
IM	39	41	4	
MM	14	24	11	

^†^Data are described by mean ± standard deviation for continuous variables.

^‡^For demographic and clinical variables nominal *P*-values were calculated by an *F*-test using ANOVA; for I148M the *P*-value was calculated by Fisher’s exact test. *P*-values for BMI, ALT, and AST remained < 0.01 after Bonferroni multiple testing correction (9 tests).

The distribution of the I148M variant in cases and controls was summarized in Table 2. In controls it was in HWE (*P*-value=0.59), which guaranteed the genotyping quality, but in cases it was out of HWE (*P*-value=0.02), which indicated its association with the disease status [32]. We previously showed the I148M variant was associated with the NAFLD affection status with the minor allele M being more frequent in cases than in controls (0.45 versus 0.31; *P*-value=1.5×10^-4^) [17]. This variant was also associated with the ultrasonography-determined steatosis degree with the M allele frequencies being 0.32, 0.54, and 0.87 in mild, moderate, and severe cases, respectively (*P*–value = 7.6×10^-8^). We further investigated the association between I148M and other clinical parameters in cases and controls, respectively (Table 2). In cases, as the copy number of M allele increased, ALT levels significantly increased (nominal *P*–value = 1.1×10^-7^, Bonferroni corrected *P*–value = 2.0×10^-6^), and AST showed a tendency of increase but not statistically significant (nominal *P*–value = 0.07), whereas LDL-C levels showed a tendency of decrease (nominal *P*–value = 4.1×10^-3^; Bonferroni corrected *P*–value = 7.4×10^-2^). None of the clinical features were significantly associated with I148M in controls.

**Table 2:** **Association between *PNPLA3 *I148M and clinical parameters in NAFLD cases and controls**^†^

**Variable**	**Cases**			***P*-value**^**‡**^	**Controls**			***P*-value**^**‡**^
	**II (n=70)**	**IM (n=84)**	**MM (n=49)**		**II (n=94)**	**IM (n=90)**	**MM (n=18)**	
Age (years)	47.6 ± 13.3	46.9 ± 13.3	45.8 ± 14.3	> 0.10	40.2 ± 12.8	42.7 ± 12.9	46.6 ± 14.7	0.04
BMI (kg/m^2^)	26.8 ± 4.4	27.1 ± 5.3	26.7 ± 5.3	> 0.10	23.0 ± 3.1	23.8 ± 3.9	24.2 ± 2.9	> 0.10
ALT (IU/L)	32.2 ± 22.5	38.2 ± 28.1	72.2 ± 59.0	1.1×10^-7^	17.4 ± 9.2	16.5 ± 7.1	17.5 ± 5.6	> 0.10
AST (IU/L)	28 ± 20.7	30.7 ± 23.8	37.5 ± 40.5	0.07	19.9 ± 4.8	19.7 ± 3.8	19.3 ± 3.6	> 0.10
GGT (IU/L)	73.1 ± 52.1	56.1 ± 58.2	52.8 ± 46.6	> 0.10	25.3 ± 17.1	24.5 ± 16.0	23.4 ± 12.9	> 0.10
Triglycerides (mmol/L)	1.9 ± 1.3	2.2 ± 1.5	2.0 ± 1.4	> 0.10	1.0 ± 0.5	1.1 ± 0.5	1.2 ± 0.4	> 0.10
LDL-C (mmol/L)	3.6 ± 1.2	3.4 ± 0.9	3.0 ± 1.0	4.1×10^-3^	2.5 ± 1.1	2.6 ± 1.1	2.8 ± 1.2	> 0.10
HDL-C (mmol/L)	1.3 ± 0.5	1.3 ± 0.7	1.4 ± 0.6	> 0.10	1.9 ± 0.8	1.9 ± 1.0	2.0 ± 1.1	> 0.10
FBG (mmol/L )	6.0 ± 1.1	6.1 ± 1.5	6.0 ± 1.1	> 0.10	5.5 ± 1.3	5.6 ± 1.4	5.9 ± 0.9	> 0.10

^†^Data are described by mean ± standard deviation.

^‡^Nominal *P*-values were calculated by fitting linear regression models, in which Age and BMI were adjusted for when testing other variables. After Bonferroni multiple testing correction (18 tests), the only significant association was between ALT levels and genotype in cases (adjusted *P*-value = 2.0×10^-6^).

**Figure 1:**
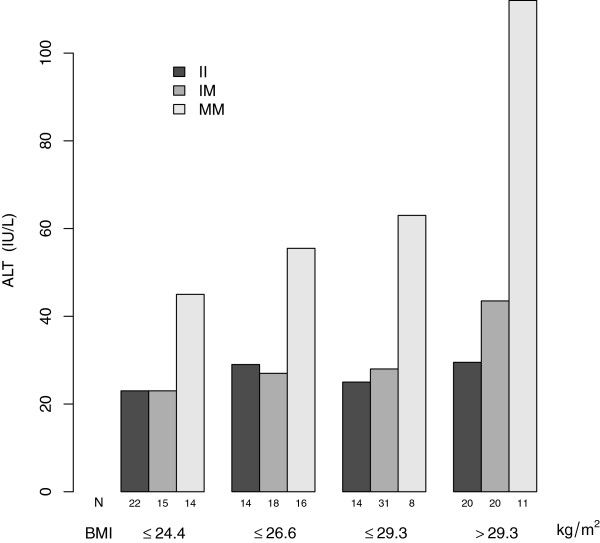
**Interaction between *PNPLA3 *I148M and BMI towards elevated ALT levels in NAFLD cases.** Cases were first stratified into quarters by BMI; in each quarter cases were further stratified by the I148M genotype, and in each group the median of ALT levels was plotted.

There was no statistically significant association between I148M and BMI in either cases or controls, or the combined sample (*P*-values > 0.10). Therefore we investigated their interaction towards ALT levels and NAFLD susceptibility assuming independence between them. Given the allele frequency of I148M variant in cases and the distributions of ALT levels and BMI in each genotypic category, the power to detect a significant interaction between I148M and BMI at the level of 0.05 was greater than 0.99 [27], and we did detect an interactive effect between them towards elevated ALT levels in cases (*P*–value = 4.4×10^-4^) by a linear regression model. Stratifying the cases into quarters by BMI, it showed the MM genotype interacted with high BMI to elevate ALT levels (Figure 1). The power to detect an intercation between I148M and BMI towards NAFLD susceptibility was estimated to be 0.12 for a case-control design [28,29] and 0.20 for a case-only design [30], respectively, and we did not detect any significant interaction given the data (*P*-values > 0.10). To detect a significant interaction at the level of 0.05 with a power of 0.8, it would need a sample size of either 2,362 cases or 1,316 cases and 1,316 controls. There was no significant interaction between I148M and BMI towards elevated AST levels or the ultrasonography-determined steatosis degree (*P*-values > 0.10).

## Discussion

It is well recognized the *PNPLA3* I148M variant predisposes susceptibility to NAFLD and it is associated with the histological severity of NAFLD. However, some studies showed it was associated with steatosis grade [33-37], whereas in two East Asian-based studies, there was no significant association between them [15,16], despite that I148M was associated with fibrosis progression in both studies. In this study we reported a positive association result between I148M and the ultrasonography-determined steatosis grade in an East Asian population.

One major limitation of this study is that the steatosis grade was assessed based on ultrasonography instead of liver biopsy, which represents the best diagnostic test for fatty liver diseases. In this study, the steatosis degree was graded by an experienced hepatologist according to established criteria [26], which showed an accuracy of 88% in the diagnosis and staging of fatty liver from a direct comparison between the pathological and ultrasonographic findings [38]. Thus the association between I148M and the ultrasonography-determined steatosis degree was unlikely false positive due to technology limitations.

Plasma liver-enzyme levels are widely used as indicators of liver damage and they are influenced by environmental and genetic factors. ALT levels were shown to be associated with the *PNPLA3* I148M variant [11,39,40], and we replicated this association in cases but not in controls. ALT levels were more variable in cases than in controls (Table 2), which partially explained the difference of the association results. We did not merge cases and controls together for an association test because of heteroscedasticity.

Although the association of *PNPLA3* with hepatic fat content and liver function is well established, its involvement in lipoprotein metabolism remains indeterminate. In this study the 148M allele was associated with reduced LDL-C levels in the NAFLD patients (nominal *P*–value = 4.1×10^-3^); when the ALT levels were adjusted for, the association became more significant (*P*–value = 3.6×10^-3^). This result was consistent with that in another large study [41], implying the association between I148M and LDL-C levels was not simply a consequence of liver function impairment. *In silico* bioinformatic analysis suggested that *PNPLA3* be involvoed in the metabolism of apoB-containing lipoproteins [41]. Although *in vitro* experiments suggested that *PNPLA3* be involved in triglycerides metabolism [11,42], there was no association between I148M and triglycerides either in normal populations [11] or in our NAFLD cases (*P*–value > 0.10). However, there were reports on their association in obese populations [25,41]. We speculate that I148M interacts with BMI / body fat distribution on triglyceride levels.

It is intriguing to investigate whether metabolic comorbidities such as obesity and insulin resistance, which are associated with the pathogenesis of NAFLD, are also influenced by the *PNPLA3* I148M variant. There was no significant association between I148M and BMI in either cases or controls, consistent with the results in general populations [11,13]. It was suggested that body fat distribution, in particular abdominal fat, be implicated in the risk of developing NAFLD [43]. There were studies showing interaction between I148M and obesity parameters (BMI and waist circumference) on ALT levels [21,24] and type 2 diabetes susceptibility [25]. In this study we observed that the MM genotype interacted with high BMI to elevate ALT levels in cases; however, their interaction towards NAFLD susceptibility was insignificant by either a case-only or a case-control study. Larger sample studies are needed to investigate whether this interaction contributes to NAFLD susceptibility and triglyceride levels.

## Competing interests

The authors declare no competing interest.

## Authors’ contributions

YL and ZT carried out clinical studies; CX and HK carried out data analysis; YL and CX were in charge of manuscript writing and project supervising. All authors read and approved the final manuscript.

## Pre-publication history

The pre-publication history for this paper can be accessed here:

http://www.biomedcentral.com/1471-2350/13/113/prepub
